# Supraphysiological Testosterone Therapy as Treatment for Castration-Resistant Prostate Cancer

**DOI:** 10.3389/fonc.2018.00167

**Published:** 2018-05-22

**Authors:** Hung-Ming Lam, Eva Corey

**Affiliations:** Department of Urology, University of Washington School of Medicine, Seattle, WA, United States

**Keywords:** supraphysiological testosterone, bipolar androgen therapy, castration-resistant prostate cancer, enzalutamide, abiraterone

Blocking androgen signaling has been the focus of treatment for advanced and metastatic prostate cancer (PC) for the past 70 years (1). First-line androgen deprivation therapy (ADT), either through surgical or medical castration (luteinizing hormone-releasing hormone agonists and antiandrogens), holds promise for PC patients; however, the disease inevitably progresses to castration resistance (2). Second-generation ADT, abiraterone acetate (AA), and enzalutamide (ENZ), have been effective for a subset of patients with castration-resistant PC (CRPC) with relatively short median survival benefits (~3–5 months) (3–5). Concerted effort in the field, including evidence from our group, clearly demonstrates a sustained AR activity in the CRPC tumors including (1) amplification of AR, (2) AR mutations, (3) expression of AR splice variants that are constitutively active, (4) altered milieu of AR coactivators and corepressors, and (5) intracrine synthesis of androgens to support CRPC progression (6, 7).

The addiction of PC to the AR signaling paradoxically creates a therapeutic vulnerability that has recently attracted increasing attention. While ADT causes regression of PC, high level of androgen can also inhibit PC progression. The concept of cancer suppression using excessive hormone therapy was introduced by earlier work from Huggins in 1940: “malignant cells can regress from too little or too much hormone” (8). In relation to PC, AR regulates proliferation as well as differentiation of prostate epithelial and cancer cells but it has not been established what conditions support one over the other. Interestingly activation of AR with excessive hormone (i.e., supraphysiological levels of testosterone; SPT) was shown to inhibit growth of CRPC *in vitro* by negative effects on proliferation and increased expression of some of the AR-regulated genes that are expressed in differentiated luminal epithelium, e.g., prostate-specific antigen. Multiple preclinical studies demonstrated that SPT inhibits growth of PC cells that express AR (9–21), with evidence suggesting that higher levels of AR might lead to more pronounced SPT effects in certain phenotypes of CRPC [reviewed in Ref. (22)]. However, AR by itself is not necessarily sufficient for the SPT-induced growth inhibition; cellular context (23) and AR-regulated transcriptome in its entirety will need to be assessed to delineate the molecular effect of SPT (24). Mechanistically, SPT-induced cell growth inhibition involves (1) cell-cycle arrest, (2) disruption of AR-mediated DNA licensing, (3) DNA damage, (4) transcriptional repression of AR and its variants, (5) transcriptional reprogramming, (6) cellular quiescence or senescence, and (7) induction of apoptosis [reviewed in Ref. (22)]. However, these effects were demonstrated exclusively in cell line models, and whether they play a significant biological role in SPT-induced tumor inhibition in patients remains to be determined.

Clinical use of testosterone (T) supplementation in PC has been limited and provided controversial results. Two older National Prostatic Cancer Project trials that used T-supplementation to normal levels with a goal to enhance the effectiveness of chemotherapy reported disappointing results (25, 26). Additional two phase I trials, which did not achieve consistent supraphysiological T levels, showed minimally reduced disease progression (27, 28). In contrast, several other studies showed that T-supplementation to normal-supraphysiological range (303−2637 ng/dl), specifically in symptomatic hypogonadal PC patients, provided prolonged disease control (as measured by sustainably low-PSA level) (29–31). In our opinion, the lack of favorable response in some of the clinical trials is, at least in part, due to the absence of a supraphysiological level of T as well as the unselected patient population.

With advanced understanding of the biology and AR involvement in CRPC progression, leveraging the active AR signaling to explore therapeutic opportunity has recently received renewed attention in clinical settings. Dr. Denmeade’s group at John Hopkins University pioneered a therapy termed “bipolar androgen therapy” (BAT) as a treatment for PC patients. With BAT treatment, PC patients receive intermittent T injections at doses shown to produce a spike in serum T to supraphysiological levels, followed by a decline to below normal at the end of a 28-day treatment cycle (32). This cycling strategy was developed based on the most common molecular hallmark of CRPC–overexpression of AR (33) and the potential growth inhibitory effect of SPT in AR-overexpressing PC. Rapidly cycling of T from SPT (~1,500 ng/dl) to below normal T levels (~150 ng/dl) was expected to blunt the adaptive changes in AR expression, thereby delaying the emergence of resistance. In these proof-of-principle BAT trials, one in CRPC showed radiographic response rates of ~50% in men (32), and one in hormone-sensitive PC showed favorable PSA responses (34). Promising results of these trials led to a new clinical trial, in which asymptomatic CRPC patients that progressed on AA or ENZ receive BAT, and after progression on BAT the patients are re-challenged with AA or ENZ. This trial aims to evaluate the efficacy of BAT in patients who progressed on secondary ADT and assess whether BAT re-sensitizes CRPC to secondary ADT. Recent data from this trial showed a PSA50 response in 9/30 ENZ-resistant patients on BAT, and, importantly, 15/21 patients who progressed on BAT showed a PSA50 response upon ENZ re-challenge (35). These results are encouraging. However, additional analyses and larger number of patients are needed to correlate tumor/radiographic vs. PSA responses in individual patients. One of the reasons is that PSA changes do not necessarily associate with tumor regression in advanced CRPC. PSA, an AR-regulated gene, is highly sensitive to AR activation/inhibition and can rise upon SPT and decline upon ADT. PSA response might not faithfully reflect radiographic responses in advanced CRPC which growth often does not rely solely on AR signaling [e.g., FGF signaling (36)].

Bipolar androgen therapy shows great clinical promise in a subset of patients. However, universal to all cancer treatment modalities, not all patients respond to this treatment and resistance to BAT develops. Therefore, there is an opportunity to improve this therapy. It is notable that a critical step in drug development, determining the optimal dosing schedule, was bypassed in the clinical development of BAT. Despite the clinical efficacy of BAT, there were by far no clinical data to support the hypotheses that cycling SPT (i.e., BAT) mitigates the development of resistance or that BAT represents the optimal mode for administering SPT. Notably, several preclinical studies have consistently demonstrated that SPT delivered on a continuous basis inhibits the growth of PC cells (13, 20, 21). While several small clinical trials of continuous T administration in men with CRPC have been carried out, they did not achieve SPT levels.

Cycling or not cycling—that is the question. While we currently do not have sufficient evidence whether BAT results in better clinical outcome than continuous SPT, it is possible that long-term continuous SPT and BAT could alter AR signaling differently. One would anticipate that continuous SPT might trigger more pronounced differentiation, potentially causing a change from a “low-T” oncogenic AR transcriptome to that of a more differentiating SPT transcriptome (24). Meanwhile, BAT might provide better efficacy if cell-cycle relicensing effects and DNA damage are the critical mechanism of action (37, 38). While BAT was associated with improved quality of life (34, 35), this effect diminished over the course of a cycle of BAT, presumably due to T levels falling below normal range. It is possible that quality of life metrics will be better with continuous SPT but there also might be increase in negative side effects. While T therapy has been reported to be generally safe, with a small subset of patients experiencing severe cardiovascular-related complications (27–32, 39–42), continuous SPT has not been tested and monitoring will be essential. Careful evaluation of effects of BAT vs. continuous SPT on tumor progression, as well as any potential health benefits or side effects will be required to make final decision.

Interestingly, cycling of ADT, intermittent ADT, has been evaluated in PC extensively since its introduction in mid-1980s. However, intermittent ADT was not found to be inferior to continuous ADT with respect to the overall survival but it was shown to improve patients’ quality of life, and therefore it is thought to be a viable option for patients who experience significant adverse effects of continuous ADT [for review see Ref. (43, 44)]. In addition, intermittent AA therapy has been recently shown to delay the development of resistance from 16.5 (continuous treatment) to 27 months (45). Whether the intermittent therapy diversifies the residual tumor clones or re-sensitize the residual clones to a therapy that formerly failed remains scientifically and clinically important.

In summary, we will need to seek answers to multiple important questions before unleashing the full potential of SPT therapy in CRPC: (1) which mode of SPT, BAT or continuous SPT, represents the optimal administration regimen for tumor growth inhibition; (2) what population of patients will benefit from SPT therapy; (3) is there a way to prolong the treatment response; and (4) what are the mechanisms of resistance, as these will be diverse in different tumor phenotypes. To address these questions, systematic preclinical trials will need to be performed, and pre-treatment and on-treatment clinical specimens will be essential to identify mechanisms of SPT action and biomarkers that predict SPT response.

## Author Contributions

H-ML and EC jointly conceptualized and wrote the manuscript.

## Conflict of Interest Statement

The authors declare that the research was conducted in the absence of any commercial or financial relationships that could be construed as a potential conflict of interest.
